# Association of Serum Leptin With Body Mass Index in Gallbladder Cancer Patients: A Pilot Study

**DOI:** 10.7759/cureus.39018

**Published:** 2023-05-15

**Authors:** Sarath Krishnan M P, Amit Gupta, Sweety Gupta, Sujata Rani, Anissa A Mirza, Bela Goyal

**Affiliations:** 1 Biochemistry, All India Institute of Medical Sciences, Rishikesh, Rishikesh, IND; 2 General Surgery, All India Institute of Medical Sciences, Rishikesh, Rishikesh, IND; 3 Radiation Oncology, All India Institute of Medical Sciences, Rishikesh, Rishikesh, IND

**Keywords:** gallbladder cancer, body mass index (bmi), tumour markers, resectability, staging, serum leptin

## Abstract

Background: Leptin has been proposed to be a link between obesity and the increased incidence of various cancers like breast cancer, colon cancer, gastric cancer, etc. The role of leptin in gallbladder cancer is largely undetermined. Moreover, no study has evaluated serum leptin levels and their correlation with clinicopathological characteristics and serum tumour markers in gallbladder cancer (GBC). Therefore, the present study was planned.

Methods: A cross-sectional study was conducted in a tertiary care hospital in Northern India after obtaining ethical approval from the institution. Forty GBC patients staged as per American Joint Committee on Cancer (AJCC) 8th staging system were recruited along with 40 healthy controls. Serum leptin was assayed by sandwich enzyme-linked immunosorbent assay (ELISA) and tumour markers (CA19-9, CEA and CA125) by Chemiluminescence. ROC, Mann Whitney U test, Linear regression and Spearman correlation was performed using Statistical Product and Service Solutions (SPSS) (IBM SPSS Statistics for Windows, Version 25.0, Armonk, NY). BMI was also assessed for both groups.

Results: Median BMI for GBC patients was 19.46 (IQR 17.61-22.36). Median serum leptin levels were significantly lower (2.09 (IQR 1.01-7.76) ng/mL) in GBC patients as compared to controls (12.32 (IQR 10.50-14.72) ng/mL). AUC was 0.84 with 100% sensitivity and 75% specificity at 7.57 ng/mL. Serum leptin was not associated with cancer stage, resectability, metastasis, liver infiltration, or tumour markers on linear regression (p=0.74, adjusted R square = -0.07). A significant positive correlation was found between BMI and serum leptin in GBC patients (p=0.00).

Conclusions: Lower BMI and relatively lean presentation of GBC patients may account for low serum leptin levels.

## Introduction

The carcinoma gallbladder is a common tumour of the biliary tract worldwide [1]. Gallbladder cancer (GBC) has a relatively poor prognosis compared to other cancers in the biliary tract due to its extensive metastatic nature, difficult anatomical position and also due to complex porto-hepato biliary system [2]. Moreover, the lack of sensitive screening tests for early detection results in delayed diagnosis and cancer ends up in the advanced stage [3,4]. Treatment for GBC patients is only by surgical resection, which is feasible in only 10% of the patients [2]. Even recurrence rates remain high in patients who have undergone surgery [3]. There is still a paucity of validated biomarkers in GBC which may help not only in early diagnosis but also determine the prognosis of GBC patients. Understanding the pathogenesis of GBC may help in identifying potential biomarkers for determining diagnosis, prognosis and potential therapeutic targets.

Obesity is a known risk factor for gallstones and GBC [1]. Obese individuals have been found to have high levels of the circulating hormone ‘Leptin’ [5]. Leptin, the product of the OB(Obesity) gene, is a 16 kDa non-glycosylated peptide hormone which is synthesized almost exclusively by adipocytes that regulate appetite and energy expenditure at the hypothalamic level [6]. Recent studies suggest that leptin plays an important role in tumorigenesis, angiogenesis and metastasis of many cancers. Leptin has been proposed to be a link between obesity and cancers in various preclinical studies. Serum leptin level has been studied with conflicting results in cancers such as breast cancer, pancreatic cancer, colon cancer, gastric cancer, etc. [6-10].

However, the expression of serum leptin and GBC has not been fully investigated, and the precise role of leptin in the development and promotion of GBC remains unknown. Therefore, the current study was done to evaluate the serum leptin concentration in GBC patients and its correlation with staging and tumour markers.

## Materials and methods

Study population

This cross-sectional study was conducted in a tertiary care hospital in Northern India after obtaining ethical approval from the institution (reference no: AIIMS/IEC/18/535). Forty clinically and radiologically suspected or histopathologically biopsy or fine needle aspiration cytology (FNAC) proven cases of GBC patients aged ≥18 years were included in the present study. Contrast-enhanced computed tomography (CECT) abdomen and pelvis was done as per the standard protocol and staging was done as per the American Joint Committee on Cancer (AJCC) eighth staging system. The resectability was determined based on the radiological findings. The involvement of the hepatic artery, portal vein, direct extension into adjacent organs, enlarged aorto-caval lymph nodes, and presence of omental or peritoneal deposits, all of which deemed the patients unresectable. Forty healthy controls were also recruited. Body mass index (BMI) was calculated for both GBC patients and controls using the formula.

Body mass index (BMI) = Weight in Kg ÷ (Height in m^2^)

(Reference range = 18.5-24.9 kg/m^2^) [11]

Serum leptin and serum tumour markers assessment

Five millilitres of venous blood was collected in plain vials from each participant. The samples were centrifuged, serum separated and stored at -80°C. Serum leptin was measured by a commercially available FDA-approved enzyme-linked immunosorbent assay (ELISA) kit (DRG®, USA) by following the manufacturer’s instructions. The lowest level of serum leptin detected by the kit is 0.7 ng/mL and the highest level detected is 100 ng/mL. All standards were run in duplicates and the standard curve was plotted (ELISA Plate reader - BioTek Eon™ High Performance Microplate Spectrophotometer, USA).

Serum tumour markers-CA19-9, CEA and CA125 were analysed by chemiluminescence assay using Siemens Advia Centaur XP analyser (Germany) by following the manufacturer’s instructions. Analysis was done after satisfactory two-level quality controls. Reference level cut-off was taken as 30.9 IU/mL, 5 ng/mL and 30.2 U/mL for CA 19-9, CEA and CA 125 respectively.

Statistical analysis

Baseline characteristics and patient clinical data were expressed in percentages. Serum leptin, tumour markers and BMI were reported as the median and interquartile range (25th percentile and 75th percentile). Shapiro Wilk normality test showed that the values are not in the normal distribution. A bivariate Mann-Whitney U test was used to compare serum leptin levels among different groups. Receiver-operating characteristics (ROC) curve analysis was performed to determine the utility of serum leptin in identifying GBC patients and to determine sensitivity and specificity at the optimal cut‑off value of serum leptin. Linear regression was performed to find the association of serum leptin with stages, resectability, tumour markers, liver infiltration and metastasis. The correlation between serum leptin and BMI in GBC patients was assessed by Spearman correlation. P values of < 0.05 were considered statistically significant. All data analysis was performed using Statistical Product and Service Solutions (SPSS) (IBM SPSS Statistics for Windows 25.0, Version, Armonk, NY).

## Results

A total of 80 samples were included in the study out of which 40 were GBC patients. Eighty per cent of GBC patients were females. The most common presenting symptom was abdominal pain (92.5%). Most of the patients presented at an advanced stage with 65% in stage IV cancer. Seventy-five per cent of patients had associated gallstones and 82.5% had liver infiltration. Eighty-five per cent of patients had unresectable disease and were subjected to palliative chemotherapy (Table 1).

**Table 1:** The clinicopathological characteristics of patients

Patient characteristics	n=40
Age (years)	53.7±12.2
Body mass index (kg/m^2^)	19.46 (IQR 17.61-22.36)
Gender: Males	8 (20%)
Gender: Females	32 (80%)
Personal History: Smoking	12 (30%)
Personal History: Alcohol	5 (12.5%)
Symptoms: Fever	19 (47.5%)
Symptoms: Jaundice	20 (50%)
Symptoms: Abdominal pain	37 (92.5%)
Gallbladder mass	36 (90%)
Gallbladder stones	30 (75%)
Liver infiltration: Absent	7 (17.5%)
Liver infiltration: Present	33 (82.5%)
Lymph nodes: Absent	7 (17.5%)
Lymph nodes: Present	33 (82.5%)
Metastasis: Absent	14 (35%)
Metastasis: Present	26 (65%)
Resectable: No	34 (85%)
Resectable: Yes	6 (15%)
TNM stage I	6 (15%)
TNM stage II	1 (2.5%)
TNM stage III	7 (17.5%)
TNM stage IV	26 (65%)

Serum leptin in GBC patients and controls

**Figure 1:**
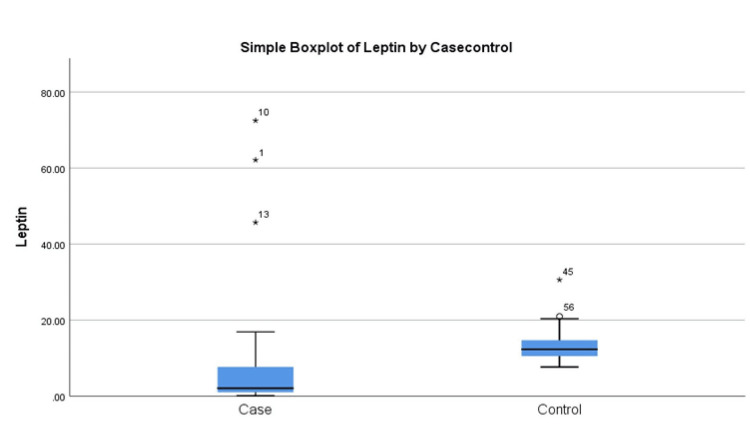
Box plot showing serum leptin distribution in GBC cases and healthy controls GBC: gallbladder cancer

The median serum leptin levels in GBC patients were 2.09 (IOR 1.01-7.76) ng/mL and were found to be significantly lower than the control group 12.32 (IQR 10.50-14.72) ng/mL in (p=0.00). The median serum leptin level in female patients (2.16 (IQR 1.06-7.76) ng/mL) is higher compared to male patients (1.64 (IQR 0.63-7.72) ng/mL) (p= 0.49) (Table 2). Figure 1 shows the box plot representing serum leptin values in GBC patients and controls.

**Figure 2:**
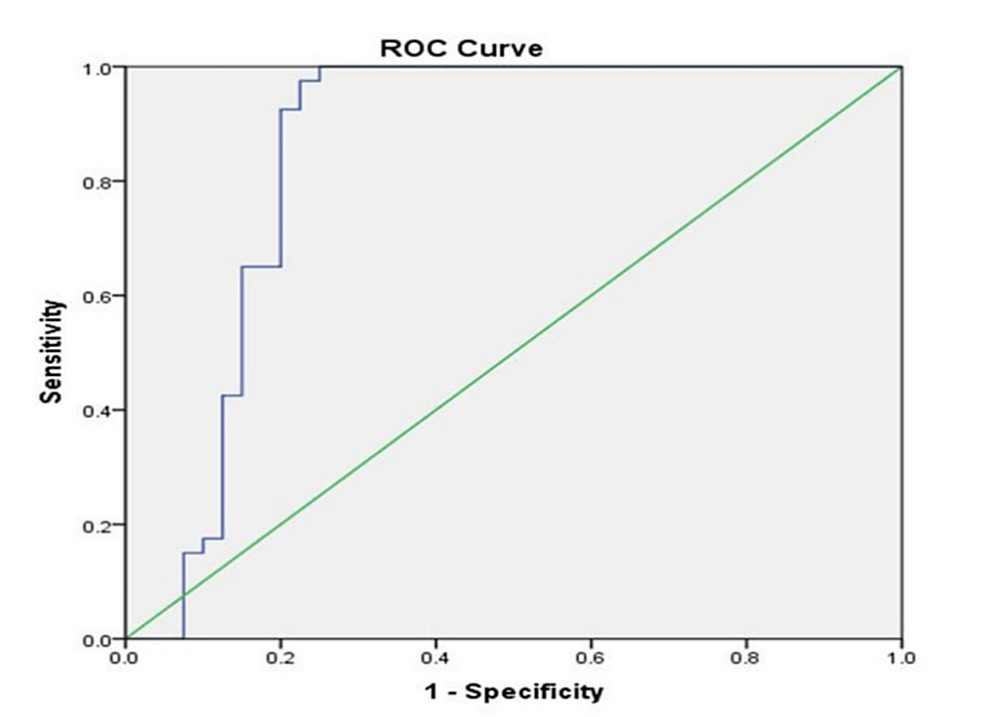
Receiver-operating characteristics curve depicting clinical utility of serum leptin value in the diagnosis of GBC patients GBC: gallbladder cancer; ROC: receiver-operating characteristics

To determine the diagnostic accuracy or usefulness of serum leptin to classify GBC patients and controls, ROC was plotted (Figure 2). An area under the curve (AUC) of 0.849 was observed indicating a high discriminatory potential of leptin in classifying GBC patients from controls. A sensitivity of 100% and specificity of 75% were observed at an optimal cut-off value of 7.578 ng/mL as determined by Youden’s index, indicating that decreased expression of serum leptin may assist in identifying cases of GBC with sufficient accuracy.

Association of serum leptin with staging, resectability and serum tumour markers in GBC

On comparing serum leptin levels in different stages of GBC, it was lower in advanced stages (stage III and stage IV) with a median value of 2.03 (IQR 1.03-7.30) ng/mL as compared to early stages (stage I and stage II) with a median of 2.29 (IQR 0.29-13.07) ng/mL. However, this was not statistically significant (p=0.83). Serum leptin levels were lower in resectable GBC patients with a median value of 1.74 (IQR 0.25-27.92) ng/mL as compared to unresectable 2.09 (IQR 1.04-7.58) ng/mL albeit non-significant (p=0.86) (Table 2).

**Table 2:** Comparison of serum leptin levels based on gender, stage and resectability GBC: gallbladder cancer

Leptin	Median (IQR) ng/mL	z value	p value
GBC patients	2.09 (1.01-7.76)	-5.36	0.00
Controls	12.32 (10.50-14.72)		
Male	1.64 (0.63-7.72)	-0.71	0.49
Female	2.16 (1.06-7.76)		
Early stage (I and II)	2.29 (0.29-13.07)	-0.23	0.83
Late stage (III and IV)	2.03 (1.03-7.30)		
Resectable cases	1.74 (0.25-27.92)	-0.18	0.86
Unresectable cases	2.09 (1.04-7.58)		

Serum tumour markers were also assessed in GBC patients. Median tumour markers values were CA 19-9: 91.04 (IQR 14.50-1200) U/mL, CEA: 3.92 (IQR 2.18-27.99) ng/mL and CA-125: 73.30 (IQR 26.47-226.22) U/mL, respectively.

No association was observed between serum leptin with staging, resectability, liver infiltration, metastasis, gallstones and tumour markers on linear regression (adjusted R square = -0.71, p=0.74) (Table 3).

**Table 3:** Association of serum leptin with clinicopathological characteristics and serum tumour markers GBC: gallbladder cancer

Predictors (Constant)	t Value	p Value
Gallstones	-1.31	0.19
Liver Infiltration	-0.87	0.38
Metastasis	1.19	0.24
GBC stages	-0.81	0.41
Resectability	-0.18	0.85
CEA	-0.50	0.61
CA19-9	-0.79	0.43
CA125	-0.66	0.51

Association of serum leptin with BMI in GBC patients and controls

BMI was found to be significantly lower in GBC patients with a median value of 19.46 (IQR 17.61-22.36) kg/m^2^ as compared to controls 23.50 (IQR 21.95-24.85) kg/m^2^ (p= 0.00).

A significant association was found between the serum leptin value of GBC patients and their BMI (p=0.00).

## Discussion

GBC is a lethal malignant disease which represents approximately 50% of all biliary tract cancers [1]. The prognosis of GBC is poor due to its aggressive tumour biology, late presentation, complicated anatomical position and advanced stage at diagnosis [3]. Locally advanced and metastatic disease treatment is with palliative chemotherapy [2]. The early stage is potentially curative with surgical resection followed by adjuvant therapy [2]. There are so many risk factors for GBC. Among them, obesity plays a pivotal role [1]. The prevalence of obesity is markedly increasing nowadays. There are several studies showing the association between obesity and various cancers [12]. In obese cancer patients, there can be changes in analytes such as adiponectin, adipokines, leptin, ceruloplasmin, etc. [8]. Among these leptin plays a key role in cancer progression. Leptin is a polypeptide produced from the obese (OB) gene, following synthesis and secretion from fat cells in white adipose tissue [13]. This peptide hormone leptin regulates food intake, body mass and reproductive function and plays a role in foetal growth, proinflammatory immune responses, angiogenesis and lipolysis [13].

Leptin has been linked to the pathogenesis of cancer by the multitude of mechanisms involving dysregulation of VEGF, JAK/STAT and AKT pathways in breast cancer, pancreatic cancer and thyroid malignancy [6]. Serum leptin has also been studied in previous studies and conflicting results have been observed in different cancers. Increased serum leptin expression has been reported in breast cancer and pancreatic cancer [8,14,15], whereas lower serum leptin levels have been observed in gastrointestinal cancers like gastric cancer and colon cancer [7,9]. The proposed mechanism for reduced expression has been explained due to insulin resistance (Arpaci F et al.) [7,9].

Although Zou Hao et al. [16] did study the expression of leptin and its functional receptor OB-Rb in GBC cancer tissue and showed a significantly higher expression in GBC tissue as compared to non-malignant tissue. However, we could not find any study assessing serum leptin expression in GBC patients.

We observed significantly lower levels of serum leptin in GBC patients compared to healthy controls. One of the plausible explanations for this could be that in the present study, most of the patients presented at an advanced stage of GBC and had a relatively lower BMI (median BMI 19.46 (IQR=17.61-22.36) kg/m^2^). Since serum leptin is well known for its relation with adipose tissue and BMI [17], a relatively lean presentation of our study group could be one of the reasons for this observation.

Previous studies on breast cancer patients that demonstrated high serum leptin levels in breast cancer also showed a positive correlation between obesity and breast cancer [6,8]. Obesity in their cohort may be responsible for the increased expression of leptin [8].

Ethnicity and geographical discordance have also been explained as one of the reasons for contradictory results of leptin among various regions [18,19]. The different techniques of measuring leptin, local leptin concentrations and leptin receptor status may also explain the heterogeneity in study findings. Polymorphisms in genes coding for leptin and its receptor may be associated with these variations [18,20].

Additionally, we observed a higher median value in stage I GBC patients compared to advanced stages suggesting a possible link of decline in serum leptin with the pathogenesis of GBC that warrants further preclinical and clinical studies exploring this facet.

In our study, no association was observed between serum leptin with staging, resectability, liver infiltration, metastasis, gallstones and tumour markers. One of the plausible explanations could be that our subjects mainly presented in advanced stages, a larger sample size with more even distribution with respect to stage may help in reaching the right conclusion.

The limitation of the current study is the lower sample size with single geographical distribution. And also, there were a smaller number of patients in the early stages due to which conclusive comment with respect to stage and resectability cannot be made. However, further preclinical and clinical studies exploring pathogenesis and polymorphism studies may help in a better understanding of the role of leptin in GBC.

This is, however, the first study demonstrating the expression of leptin in sera of GBC patients.

## Conclusions

Serum leptin is well known for its relation with adipose tissue and BMI, a relatively lean presentation of GBC patients may account for significantly low levels of leptin in sera of GBC patients and may be associated with loss of adipose tissue in GBC patients.

## Appendices

List of abbreviations

AKT pathway - Protein kinase B pathway

AJCC - American Joint Committee on Cancer

AUC - Area under the curve

BMI - Body mass index

CA 19-9 - Cancer antigen 19-9

CA 125 - Cancer antigen 125

CEA - Carcinoembryonic antigen

CECT - Contrast-enhanced computed tomography

ELISA - Enzyme-linked immunosorbent assay

FNAC - Fine needle aspiration cytology

GBC - Gallbladder cancer

IQR - Inter quartile range

JAK/STAT - Janus kinase/signal transducers and activators of transcription

OB gene - Obesity gene

ROC - Receiver-operating characteristics

VEGF - Vascular endothelial growth factor
